# Comparative Proteomic Analysis Provides New Insights into the Molecular Basis of Thermal-Induced Parthenogenesis in Silkworm (*Bombyx mori)*

**DOI:** 10.3390/insects14020134

**Published:** 2023-01-28

**Authors:** Jine Chen, Xin Du, Xia Xu, Sheng Zhang, Lusong Yao, Xiuling He, Yongqiang Wang

**Affiliations:** 1 Institute of Sericulture and Tea, Zhejiang Academy of Agricultural Sciences, Hangzhou 310021, China; 2 Proteomics and Metabolomics Facility, Institute of Biotechnology, Cornell University, Ithaca, NY 14853, USA

**Keywords:** *Bombyx mori*, parthenogenesis, thermal induction, proteome, iTRAQ, PRM

## Abstract

**Simple Summary:**

Parthenogenesis is a reproductive mode by which an oocyte goes into development without fertilization, which is common in insects and plays an important role in evolution, sociality, and ecology. However, the molecular mechanism of parthenogenesis in insects remains to be expanded. The silkworm *Bombyx mori* is an economically important insect. The artificial parthenogenesis induced in vitro using thermal stimuli has been well used in silkworm breeding as a sex control method. A better understanding of parthenogenesis and its regulatory mechanisms could benefit sericultural production. Meanwhile, silkworm provides a good material for parthenogenesis development research as a lepidopteran model. In this study, iTRAQ-based quantitative proteomics were carried out to compare the protein expression profile between a fully parthenogenetic line (PL) and its parent amphigenetic line (AL). We identified 274 increased abundance proteins and 211 decreased abundance proteins before thermal induction, and 97 increased abundance proteins and 187 decreased abundance proteins after thermal induction in PL. Function analysis uncovered the molecular signatures underlying the two different reproductive modes, and the proteins and pathways involved in a meiosis process response to thermal stimuli. The results provide new insights into the molecular mechanism underlying silkworm parthenogenesis.

**Abstract:**

Artificial parthenogenetic induction via thermal stimuli in silkworm is an important technique that has been used in sericultural production. However, the molecular mechanism underlying it remains largely unknown. We have created a fully parthenogenetic line (PL) with more than 85% occurrence and 80% hatching rate via hot water treatment and genetic selection, while the parent amphigenetic line (AL) has less than 30% pigmentation rate and less than 1% hatching rate when undergoing the same treatment. Here, isobaric tags for relative and absolute quantitation (iTRAQ)-based analysis were used to investigate the key proteins and pathways associated with silkworm parthenogenesis. We uncovered the unique proteomic features of unfertilized eggs in PL. In total, 274 increased abundance proteins and 211 decreased abundance proteins were identified relative to AL before thermal induction. Function analysis displayed an increased level of translation and metabolism in PL. After thermal induction, 97 increased abundance proteins and 187 decreased abundance proteins were identified. An increase in stress response-related proteins and decrease in energy metabolism suggested that PL has a more effective response to buffer the thermal stress than AL. Cell cycle-related proteins, including histones, and spindle-related proteins were decreased in PL, indicating an important role of this decrease in the process of ameiotic parthenogenesis.

## 1. Introduction

Parthenogenesis is a reproductive mode without fertilization. It is a very common, naturally occurring phenomenon in insects [1,2,3,4]. Parthenogenesis is frequently selected in insects for their low dispersal ability and short adult life, which may generate situations of mate limitation in females [5]. However, nearly all insect orders can reproduce parthenogenetically to some degree [6,7]. In nature, insects can reproduce by cyclical parthenogenesis (CP) or obligate parthenogenesis (OP), and both usually coexist in the same species [8,9,10]. Infectious parthenogenesis via parthenogenesis-inducing Wolbachia has also been demonstrated in insects [11]. These studies suggest that the mechanisms mediating the parthenogenesis vary among species and are driven by various environmental cues. Parthenogenesis can also be induced artificially via chemical and physical stimuli. Artificial parthenogenesis has been reported in plants [12], silkworm [13], fishes [14], and murine [15], as it is a technique of great practical significance in breeding and other practical goals [16,17].

The silkworm *Bombyx mori* is an economically important insect that is used for the production of silk; it is also a lepidopteran model organism for research in molecular biology since its genome has been completely sequenced [18,19,20,21]. Female silkworms typically reproduce sexually and only occasionally via parthenogenesis. Parthenogenesis can be induced in silkworms via hot water treatment at 46 °C for 18 min [22]. This parthenogenetic activation method has been used in silkworm breeding, as it solves the technically complex problem of preparing interbred hybrids without the need to separate the females and males anymore [13,23].

We have created parthenogenetic lines (PLs) from amphigenetic lines (ALs) through generations of selection via the thermal inductive method. The parthenogenetic capacity of PL was superior to that of AL after more than 20 generations of selection with the incidence of parthenogenesis greater than 80% [24]. The majority of the unfertilized eggs of PLs completed their development successfully when their development was induced via thermal treatment, while only a limited part of the unfertilized eggs of AL will successfully develop when subjected to the same thermal-inducing parthenogenetic progress. Therefore, the comparison between PL and its parent AL would provide a useful model for exploring the mechanism of parthenogenesis. The comparison studies between different reproductive modes, such as CP individuals and OP ones, have been reported to uncover the mechanisms underlying the transition from sexual to parthenogenetic reproduction [25,26,27,28].

Based on the cytological mechanism, parthenogenesis can be classified as apomictic parthenogenesis (i.e., ameiotic parthenogenesis, AMP), in which genetically identical progeny are produced, and automictic parthenogenesis (i.e., meiotic parthenogenesis, MP), in which progeny produced are not genetically identical. The thermal parthenogenesis of silkworm is AMP which allows to obtain isogenic parthenogenetic progeny of only female sex [13]. Although the cytological mechanism underlying thermal parthenogenesis of silkworm has been described, little is known regarding the molecular mechanism of silkworm AMP.

High-throughput sequencing has been used to generate a silkworm transcriptome of a thermal-induced PL and its parent AL. Comparative analysis revealed that the DEGs are involved in reproduction, chorion formation, female embryogenesis, and cell development [24]. However, research at the protein level is critically important for filling the knowledge gap on the molecular mechanism of parthenogenesis, as proteins are the main executors of the cell function. Moreover, the oocyte and early embryo development is likely regulated by control of the protein abundance, activity, or posttranslational modifications for lack of transcription [29]. In the present study, we employed the isobaric tags for relative and absolute quantitation (iTRAQ) labeling quantitative proteomics approach to detect the proteome changes between PL and its parent AL before and after thermal induction. Some selected differential abundance proteins (DAPs) were further confirmed by Parallel Reaction Monitoring (PRM). The comparative data would shed light on the molecular basis of different parthenogenetic capacity between PL and AL, and the process of transition from meiosis to parthenogenesis.

## 2. Materials and Methods

### 2.1. Experimental Silkworm Eggs

The silkworm PL Wu14 and its parental AL 54A were maintained in the Sericulture and Tea Research Institute of the Zhejiang Academy of Agricultural Sciences. Strain 54A is an important Japanese AL that reproduces by sexual mating. Wu14 originates from the sexually reproducing strain 54A and reproduces parthenogenetically with hot water induction (46 °C, 18 min), with genetic selection with high parthenogenetic incidence [24,30]. In this investigation, Wu14 experienced 26 generations of selection. The PL and AL silkworms were raised and treated as Liu et al. described [24]. Unfertilized eggs were dissected from virgin moths of PL Wu14 and AL 54A at the age of 11 h after eclosion. Each sample had three biological replicates, and each replicate was a mixed sample containing eggs from 14 female silkworm moths. Each replicate was divided into three parts: eggs of part one were immediately frozen in liquid nitrogen after being washed and dried, then stored at −80 °C for later protein extraction, and recorded as Wu14 un-induced (Wu14_UI) eggs and 54A un-induced (54A_UI) eggs; eggs of part two were thermally treated in a 46 °C water bath for 18 min with a recovery period of 3 min at 25 °C water bath, then quickly dried and frozen by liquid nitrogen, then stored at −80 °C for later protein extraction, and recorded as Wu14 thermal-induced (Wu14_TI) parthenogenetic eggs and 54A thermal-induced (54A_TI) eggs; eggs of part three were used to observe the parthenogenetic incidence by calculating the pigmentation and hatching rate. The thermal-induced eggs were kept at 16 °C, 80% RH for 3d, then diapause was eliminated by pickling, and they were incubated in 25 °C, 85% RH for hatching; the pigmentation rate was counted six days after thermal induction, and hatching rate was counted three days after hatching.

### 2.2. Egg Protein Extraction, Digestion and iTRAQ Labeling

**Figure 1:**
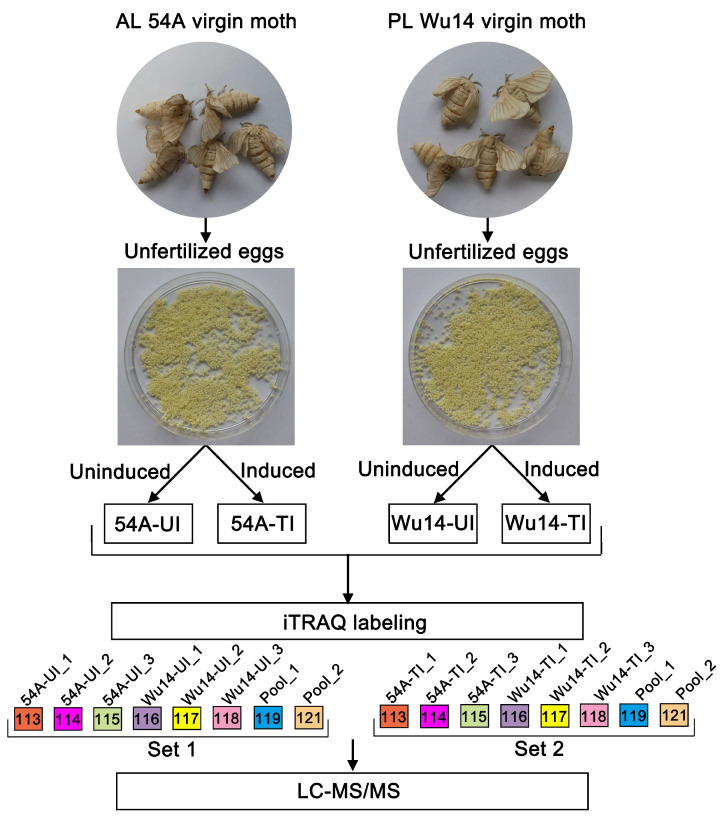
A schematic workflow of iTRAQ-based quantitative proteomics used in this study.

Proteins from 4 silkworm egg samples (Wu14_UI, 54A_UI, Wu14_TI, 54A_TI) with 0.1 g eggs for each biological replicate were extracted in a lysis containing 7M Urea, 2M Thiourea, 4% CHAPS, 1% DTT at a volume ratio of 1:10. The homogenates were centrifugated at 15,000 rpm for 10 min at 4 °C. The protein concentration was determined by Bradford assay with bovine serum albumin as a standard, and further checked on an SDS-PAGE along with a series of amounts of *E. coli* lysates (2.5, 5, 10, 20 µg/lane). In total, 100 μg proteins of each sample were reduced in 100 μL solution containing 10 mM dithiothreitol at 37 °C for 1 h. Cysteine residues were blocked with 55 mM iodoacetamide for 45 min in the dark. The proteins were precipitated by iced acetone overnight and then digested using a trypsin (Promega, Madison, WI, USA) to substrate ratio of 20:1 at 37 °C overnight. The digested peptide was reconstituted with 0.5 M Triethylammonium biocarbonate (TEAB) solution. Proteins were identified and quantified at the Cornell Proteomics Facility, using the technique of iTRAQ-based comparative proteomics analysis. A schematic workflow of iTRAQ-based quantitative proteomics is shown in Figure 1. The peptides were labeled with iTRAQ 8-plex reagents according to the instructions of the manufacturer. One set included triplicate 54A (UI) and W14 (UI) samples and the other set contained 54A (TI) and Wu14 (TI), respectively. In each set, 113-tags,114-tags,115-tags were used for AL 54A triplicate, and 116-tags, 117-tags, and 118-tags for PL Wu14 triplicate. For normalization between the two sets, 119-tags and 121-tags for a mix of all 6 samples in duplicate (pool_1 and pool_2, as shown in Figure 1) were used. After labeling, the samples with each tag were checked by nanoLC-MS/MS, making sure successful labeling was achieved. Then, the labeled samples in Set 1 and Set 2 were pooled, respectively, and subjected to high pH reverse phase fractionation.

### 2.3. Liquid Chromatography and Tandem Mass Spectrometry (LC-MS/MS)

The High pH reversed-phase chromatography was performed by Ultimate3000 MDLC platform with the built-in micro fraction collection option in its autosampler and UV detection (Dionex, Sunnyvale, CA). The iTRAQ 8-plex tagged peptides were reconstituted in 20 mM ammonium formate (NH4FA) and loaded onto an XTerra MS C18 column (3.5 mm, 2.1 × 150 mm, Waters Corp, Milford, MA, USA) with buffer A (pH 9.5) and 80% acetonitrile (ACN)/20% 20 mM NH4FA as buffer B. The LC was performed using a gradient from 10–45% of buffer B in 30 min at a flow rate 200 µL/min. Forty-eight fractions were collected at 1 min intervals and pooled into a total of 12 fractions based on the UV absorbance at 214 nm and with multiple fraction concatenation strategy [31]. All of the fractions were dried and reconstituted in 2% ACN/0.5% FA followed by a nano C18 reverse phase HPLC separation coupled with an LTQ-Orbitrap Elite (Thermo Fisher Scientific, Waltham, MA, USA) mass spectrometer equipped with nano ion source using high energy collision dissociation (HCD). The MS/MS analysis was conducted as previously reported [31].

### 2.4. Protein Identification and Quantification

The raw MS/MS data were converted into MGF format by Proteome Discoverer 1.3 (Thermo Fisher Scientific, Waltham, MA, USA), and the exported MGF files were searched using Mascot version 2.3.02 (Matrix Science, London, UK) against a combined database (25,329 sequences) downloaded from NCBI reference sequences database (ftp://ftp.ncbi.nlm.nih.gov/refseq/release/, (accessed on 28 June 2018)) and (http://silkworm.big.ac.cn/jsp/download.jsp, (accessed on 28 June 2018)). Several parameters in Mascot were set for peptide searching, including iTRAQ 8-plex for quantification; trypsin was specified as cleavage enzyme, with a tolerance of one missed trypsin cleavage, fragment mass tolerance was set at 0.1 Da, and peptide mass tolerance was set at 20 ppm. Carbamidomethyl (C), iTRAQ 8-plex (N-term), and iTRAQ 8-plex (K) were set as fixed modification, oxidation (M) and iTRAQ 8-plex (Y) as variable modifications.

For protein quantification, iTRAQ-8-plex was selected in Mascot (Matrix Science, UK). iQUANT [32] was used for quantitatively analyzing the labeled peptides with isobaric tags. To assess the confidence of peptides, the PSMs were pre-filtered at a PSM-level FDR of 1%. Then, based on the “simple principle” (the parsimony principle), identified peptide sequences were assembled into a set of confident proteins. In order to control the rate of false-positive at protein level, a protein FDR at 1% [33] was also estimated after protein inference (protein-level FDR ≤ 0.01). The protein quantification process included the following steps: protein identification, tag impurity correction, data normalization, missing value imputation, protein ratio calculation, statistical analysis, and results presentation. Both *p* < 0.05 by t test and the fold changes either >1.2-fold or <0.83-fold were used to determine the significance of DAPs. We used coefficient of variation (CV), which is defined as the ratio of the standard deviation (SD) to the mean, to evaluate reproducibility. All proteins were identified with a 95% confidence limit and <1% false discovery rate (FDR) with at least one unique peptide.

### 2.5. Bioinformatics Analysis

The gene ontology (GO) annotation and pathway analyses of the DAPs using the GO (http://www.geneontology.org/, (accessed on 18 July 2018)), KEGG (http://www.genome.jp/kegg/, (accessed on 18 July 2018)), and eukaryotic orthologous groups (KOG) databases (www.ncbi.nlm.nih.gov/research/cog/, (accessed on 18 July 2018)) were performed by BLASTP, and a hypergeometric test was used to define significantly enriched GO terms and KEGG pathways. The *p* value < 0.05 was considered as significant. The protein–protein interaction (PPI) network analysis of DAPs was performed by the STRING database (STRING 11.0), and the fasta files containing the target protein sequences were matched. More than 90% of the proteins with the same identity were selected for constructing the regulatory network. The subcellular location was predicted using prediction soft wolfpsort [34].

### 2.6. Parallel Reaction Monitoring (PRM) for Target Conformation

Ten DAPs from the most enriched pathways and five randomly selected DAPs were further quantified and confirmed using targeted LC-PRM-MS. To be consistent with the iTRAQ experiment, protein extraction and tryptic digestion were performed in the same way as in the iTRAQ. The digested samples were added with 50 fmol of β-galactosidase for data normalization. PRM analyses were performed on a Q Exactive HFX mass spectrometer (Thermo Fisher Scientific, San Jose, CA, USA) equipped with UltiMate 3000 UHPLC system (Thermo Fisher Scientific, San Jose, CA, USA). The dried peptide samples were reconstituted with mobile phase A (2% ACN, 0.1% FA). Separation was performed by Thermo UltiMate 3000 UHPLC. The samples were first enriched in trap column and desalted, and then entered a self-packed C18 column (75 μm internal diameter, 3 μm column size, 25 cm column length) and were separated at a flow rate of 300 nL/min by the following effective gradient: 0~5 min, 5% mobile phase B (98% ACN, 0.1% FA); 5~45 min, mobile phase B linearly increased from 5% to 25%; 45~50 min, mobile phase B increased from 25% to 35%; 50~52 min, mobile phase B rose from 35% to 80%; 52~54 min, 80% mobile phase B; 54~60 min, 5% mobile phase B. The peptides separated by liquid phase chromatography were ionized by a nanoESI source and then passed to a tandem mass spectrometer Q-Exactive HF X (Thermo Fisher Scientific, San Jose, CA, USA) for full MS + PRM mode detection. The main parameters were set: ion source voltage was set to 1.9 kV, MS1 mass spectrometer scanning range was 350~1500 m/z; resolution was set to 60,000; MS2 resolution was 15,000; and the MS2 starting m/z was fixed at 100. The AGC was set to: MS1 3E6, MS2 1E5.

Skyline software was used to integrate the raw file generated by Q-Exactive HF X (Thermo Fisher Scientific, San Jose, CA, USA), and an iRT strategy was used to define a chromatography of a given peptide against a spectral library. All transitions for each peptide were used for quantitation unless interference from the matrix was observed. A spike of β-galactosidase was used for label-free data normalization. The MSstats was used with a linear mixed-effects model. All proteins with a *p* value below 0.05 and a fold change larger than 1.2 or smaller than 0.83 were considered significant.

## 3. Results

### 3.1. Incidence of Parthenogenesis

**Figure 2:**
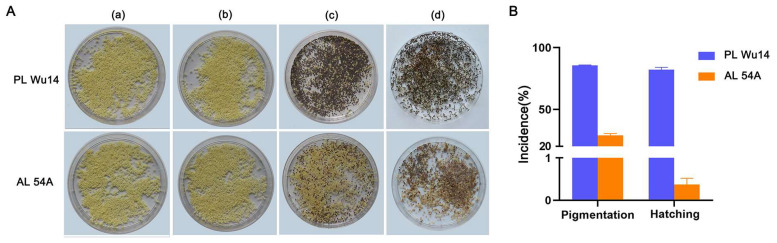
Incidence of parthenogenesis in the unfertilized eggs of parthenogenetic line (PL) Wu14 and its parent amphigenetic line (AL) 54A. (**A**) The phenotype of the eggs in different developmental stages. (**a**) The unfertilized eggs without treatment. (**b**) The unfertilized eggs right after thermal treatment. (**c**) The unfertilized eggs six days after thermal treatment. (**d**) The unfertilized eggs three days after hatching (i.e., 15 days after thermal treatment). (**B**) The incidence of parthenogenesis. The pigmentation rate is the ratio of the number of pigmented eggs to the total number of eggs treated. The hatching rate is the ratio of the number of eggs that hatched into larvae to the total number of eggs treated. The pigmentation rate was calculated six days after thermal treatment. The hatching rate was determined three days after hatching. The value of each fraction was the mean ± standard deviation (*n* = 3). Pigmentation rate and hatching rate data are the averages of three replicates (each replicate contains 0.2 g of eggs obtained from mixed samples containing 14 female silkworm moths).

The frequency of haploid occurrence of PL Wu14 and AL 54A after thermal induction was assessed by pigmented eggs and hatched larvae. Eggs lost at the early developmental stages after parthenogenetic activation preserved yellow color, whereas those arrested at metaphase I began to develop and acquired a dark color on the third day due to serose membrane pigmentation [35]. Therefore, pigmentation of the eggs (silkworm eggs shift from yellow to grey color) was used as an indicator of successful thermal parthenogenesis. However, not all pigmentated eggs will hatch successfully. In PL Wu14, pigmentation incidence and hatchability were 85.69 ± 0.62% and 81.99 ± 2.08%, respectively, while in AL 54A, these were only 28.98 ± 1.89% and 0.50 ± 0.26%, respectively (Figure 2 and Supplementary Table S1).

### 3.2. Protein Profile and DAPs from Quantitative Proteomics Analysis

With unique peptides ≥ 1 and 1% FDR, a total of 1426 proteins were identified in comparison of Wu14_UI vs. 54A_UI, and 1516 proteins in comparison of Wu14_TI vs. 54A_TI. In total, 1415 and 1496 proteins were reliably quantified (detected in ≥2 biological replicates). The proteins were identified with high peptide coverage, of which 54.63% and 55.41% were identified with more than 8% sequence coverage before thermal induction and after induction, respectively, and 37.53% and 40.17% proteins were identified with three or more peptides. The details of the peptides matched and quantification data for these identified proteins are provided in Supplementary Tables S2 and S3.

**Figure 3:**
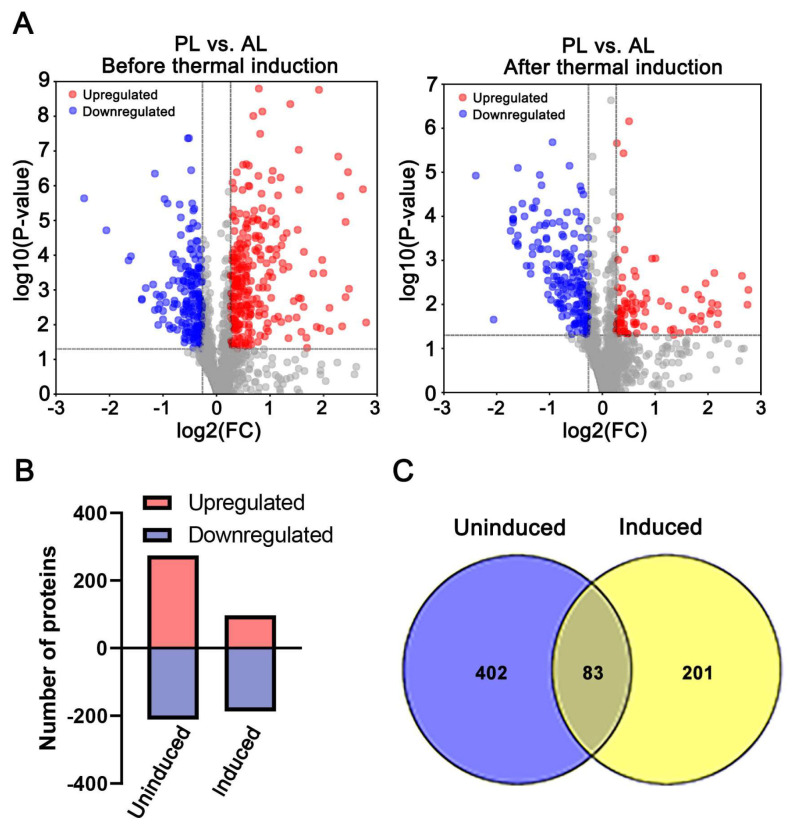
The number and distribution of differential abundance proteins (DAPs) between PL and AL. Comparison 1 and comparison 2 were PL vs. AL before and after thermal induction, respectively. (**A**) The volcano plot of identified proteins. The red dot indicates the proteins with increased abundance, the blue one indicates the proteins with decreased abundance, and the gray dot indicates the non-significantly changed proteins. (**B**) Quantification of the increased and decreased proteins in the two pairwise comparisons (uninduced and induced) between PL and AL. (**C**) Venn-diagram showing the DAPs between the two pairwise comparisons.

In order to obtain proteome profile patterns of PL and AL before and after thermal induction, DAPs were analyzed with threshold at FC ≥ 1.2 or FC ≤ 0.83, and *p* ≤ 0.05 cutoff for DAPs identification based on iTRAQ result. In total, 485 DAPs, including 274 proteins with an increased abundance in PL and 211 with decreased abundance, were identified before thermal induction (Figure 3A,B, Supplementary Table S4), and 284 DAPs, including 97 proteins with increased abundance and 187 ones with decreased abundance, were identified after thermal induction (Figure 3A,B, Supplementary Table S5). There are 83 proteins commonly found in both pairwise sets of data (Figure 3C, Supplemental Table S6). Among those overlapping DAPs, 50 proteins showed significantly different profile patterns between the two comparisons.

### 3.3. Subcellular Localization of DAPs

**Figure 4:**
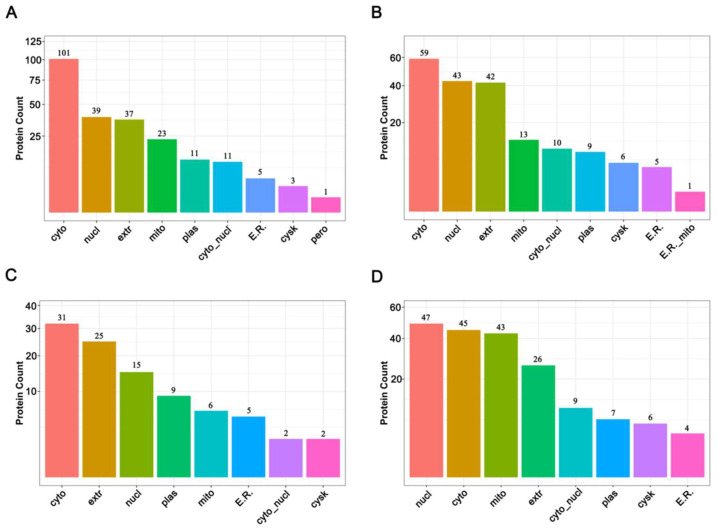
Subcellular location of DAPs before and after thermal induction. (**A**) Subcellular location of increased abundance proteins before thermal induction; (**B**) Subcellular location of decreased abundance proteins before thermal induction; (**C**) Subcellular location of increased abundance proteins after thermal induction; (**D**) Subcellular location of decreased abundance proteins after thermal induction.

The subcellular locations of proteins are closely related to their functions [36]. Before thermal induction, 231 increased abundance proteins and 188 decreased abundance proteins were finally mapped on nine different subcellular locations, and after thermal induction, 96 increased abundance proteins and 187 decreaced abundance proteins were mapped on eight different subcellular locations. The results showed that DAPs were mainly located in cytoplasm, nucleus, extracellular, and mitochondria before thermal induction (Figure 4A,B, Supplementary Tables S7 and S8), while after thermal induction, the top four locations for increased abundance DAPs were cytoplasm, extracellular, nucleus, and plasma membrane (Figure 4C, Supplementary Table S9); for decreased abundance proteins, they were nucleus, cytoplasm, mitochondrion, and extracellular (Figure 4D and Supplementary Table S10). The events of meiosis take place in the nucleus. The results of subcellular location demonstrate that among the decreased abundance DAPs, most are located in the nucleus, while only 15 nucleus proteins were increased in abundance, suggesting that the ameiosis process is a result of a decrease in some meiosis-related events. Furthermore, many mitochondria-resident proteins decreased in abundance after thermal stimuli. This may be a response to the thermal stress.

### 3.4. Expression Pattern of DAPs Associated with Heat Shock, Cell Cycel and Cytoskeleton

To investigate the DAPs with possible important roles in the process of thermal- induced ameiotic parthenogenesis. We first characterized the heat shock response in the PL and AL after thermal stimuli. A total of 17 HSPs were identified, including HSP83, HSP70, HSP60, and small HSPs; only three HSPs showed differential abundance, among them, Hsp70 and Hsc were increased in abundance and Hsp 21.4 was decreased in abundance, indicating that Hsp70 and Hsc may have a role in reponse to thermal stimuli and regulating the subsequent ameiotic process of silkworm parthenogenesis.

Subsequent to thermal induction, most of the unfertilized eggs of the PL will undergo an ameiotic process to develop by a parthenogenetic reproduction mode. We then examined the expression pattern of cell cycle-related proteins (Table 1). It was found that most of those cell cycle-related proteins were decreased in abundance in PL, including histones (histone H2A, H2B-like, histone H3-like, histone H4, and histone H1-like), all showed a decrease in PL. The cytological analysis revealed abnormal arrangement of spindles involved in the ameiotic process [13]. The proteome data showed that microtubule-associated protein futsch that controls the stability of microtubules was decreased in abundance in PL. Three actin proteins were also decreased in the PL, including alpha-actinin (sarcomeric isoform X1), actin(muscle-type A1), and actin(muscle-type A2). The decrease with the largest fold changes occurred in nesprin, tropomyosin, troponin I, and myosin in the PL (Table 2).

### 3.5. Functional Enrichment Analysis of DAPs

#### 3.5.1. GO Enrichment Analysis of DAPs

Significantly enriched GO terms in three categories including molecular function, cellular component, and biological process were obtained according to the criterion *p*-value < 0.05. The results showed that the increased abundance proteins before thermal induction were significantly enriched in ribosome, intracellular ribonucleoprotein complex, ribonucleoprotein complex, and non-membrane-bounded organelle in the cellular component category; structural constituent of ribosome, structural molecule activity, rRNA binding, and oxidoreductase activity in the molecular function category; and translation, peptide biosynthetic process, amide biosynthetic process, and gene expression in the biological process category (Table 3, Supplementary Table S11). Decreased abundance proteins before thermal induction were significantly enriched in troponin complex, striated muscle thin filament, myofibril, and sarcomere in the cellular component category; protein heterodimerization activity, structural constituent of chorion, GTPase activator activity, and enzyme activator activity in the molecular function category; and multicellular organism development, multicellular organismal process, cell development, and developmental process in the biological process category (Supplementary Table S12).

Increased abundance proteins after thermal induction were significantly enriched in external encapsulating structure, chorion, and cell periphery in the cellular component category; structural constituent of chorion, structural molecule activity, transferase activity, and transferring glycosyl groups in the molecular function category; epithelial cell development, columnar/cuboidal epithelial cell differentiation, columnar/cuboidal epithelial cell development, and chorion-containing eggshell formation in the biological process category (Table 4, Supplementary Table S13). Meanwhile, decreased abundance proteins were significantly enriched in mitochondrion, mitochondrial envelope, proton-transporting ATP synthase complex, and mitochondrial membrane in the cellular component category; electron transfer activity, ion transmembrane transporter activity, cation transmembrane transporter activity, and inorganic molecular entity transmembrane transporter activity in the molecular function category; and ATP biosynthetic process, energy-coupled proton transport, down electrochemical gradient, ATP synthesis-coupled proton transport, and oxidation-reduction process in the biological process category (Supplementary Table S14).

#### 3.5.2. KEGG Enrichment Analysis of DAPs

**Figure 5:**
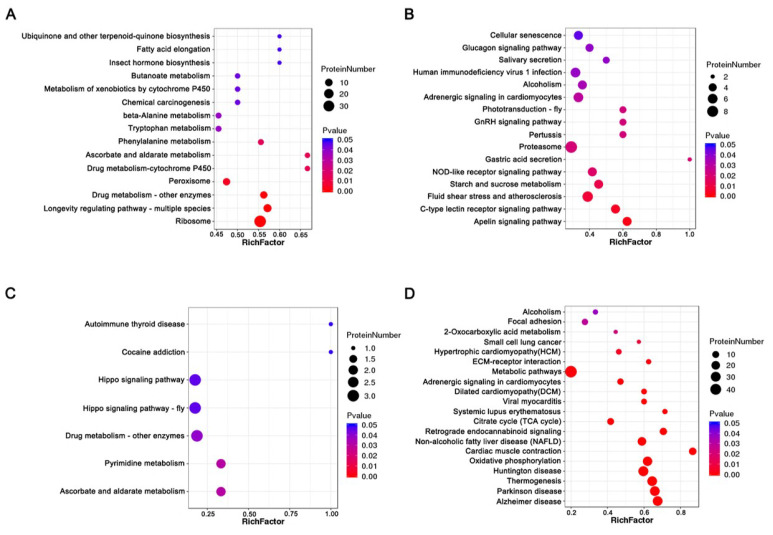
Bubble charts of KEGG pathway enrichment analysis of DAPs before and after thermal induction. (**A**) The enriched KEGG pathways for increased abundance proteins before thermal induction. (**B**) The enriched KEGG pathways for decreased abundance proteins before thermal induction. (**C**) The enriched KEGG pathways for increased abundance proteins after thermal induction. (**D**) The enriched KEGG pathways for decreased abundance proteins after thermal induction.

KEGG pathway enrichment analysis showed that increased abundance and decreased abundance proteins were significantly enriched in fifteen and sixteen KEGG pathways before thermal induction, respectively. The increased abundance proteins were most significantly enriched in ribosome, longevity-regulating pathway, drug metabolism, and metabolism pathways, such as ascorbate and aldarate metabolism, phenylalanine metabolism, tryptophan metabolism, and beta-alanine metabolism (Figure 5A, Supplementary Table S15). Decreased abundance proteins were most significantly enriched in starch and sucrose metabolism, NOD-like receptor signaling pathway, proteasome, GnRH signaling pathway, and phototransduction (Figure 5B, Supplementary Table S16). After thermal induction, seven and twenty KEGG pathways corresponding to increased abundance and decreased abundance proteins were significantly enriched. The increased abundance proteins were most significantly enriched in ascorbate and aldarate metabolism, pyrimidine metabolism, drug metabolism, and Hippo signaling pathway (Figure 5C, Supplementary Table S17). Decreased abundance proteins were most significantly enriched in thermogenesis, oxidative phosphorylation, citrate cycle, metabolism pathway, and ECM–receptor interaction (Figure 5D, Supplementary Table S18). Strikingly, a total of 29 thermogenesis-related proteins were decreased in abundance in PL. These proteins include NADH dehydrogenase of complex I; succinate dehydrogenase of complex II; cytochrome c reductase and cytochrome oxidase of complex III and complex IV; and F-type ATPase of complex Ⅴ. The maps of significant enriched pathways are provided in Supplementary Figures.

**Figure 6:**
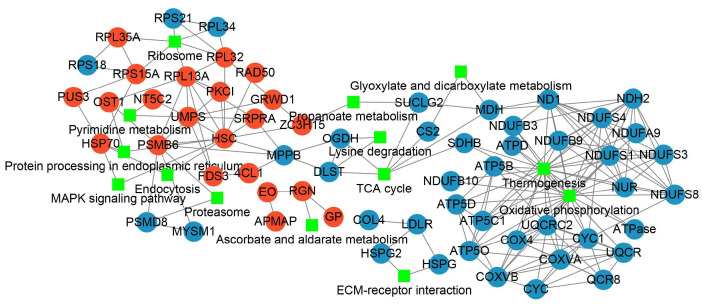
The protein–protein interaction analysis for DAPs after thermal induction. Red dot indicates the increased abundance protein, blue dot indicates the decreased abundance protein, and the green rectangle indicates the KEGG pathway involved.

To clarify the responses of PL unfertilized eggs to thermal stimuli and the trigger of parthenogenetic development, PPI was characterized for further revealing the inter-relationships and the profile patterns of the DAPs after thermal induction (Figure 6, Supplementary Table S19). A total of 21 increased abundance proteins and 41 decreased abundance proteins were respectively mapped to the PPI network. The enriched KEGG pathways being involved by more than two DAPs were exhibited. Through the interaction network displayed by Cytoscape [37], we found that heat shock cognate protein (HSC) was the node of the highest degree which interacted with other DAPs with increased abundance, including DNA repair protein RAD50 (RAD50), heat shock protein 70 (HSP70), proteasome subunit beta type-6 (PSMB6), protein kinase C inhibitor (PKCI), uridine 5’-monophosphate synthase (UMPS), glutamate-rich WD repeat-containing protein 1 (GRWD1). The DAPs with increased abundance were mainly involved in the pathways of ribosome, pyrimidine metabolism, protein processing in endoplasmic reticulum, endocytosis, proteosome, MAPK signaling pathway, and ascorbate and aldarate metabolism. For the decreased abundance proteins, thermogenesis and oxidative phosphorylation linked the largest number of proteins.

### 3.6. Validation of Protein Abundance Changes by PRM

**Figure 7:**
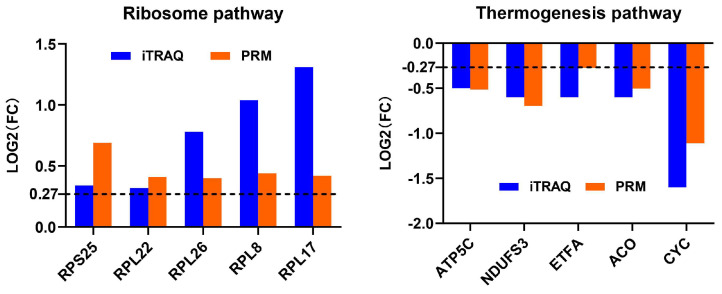
Profile patterns of differential abundance proteins (DAPs) selected from the most enriched pathway before and after thermal induction using parallel reaction monitoring (PRM) validation. Dotted lines at 0.27 represents 1.2-fold (for increased abundance) and −0.27 represents 0.83-fold (for decreased abundance).

Although we made every effort to ensure that our statistical and bioinformatic analyses in iTRAQ proteomics datasets were robust, we conducted PRM analysis on 10 DAPs from the most enriched pathways before and after thermal induction to further validate the results from the iTRAQ analysis (Figure 7, Table 5). We also randomly selected five DAPs (e.g., low molecular mass 30 kDa lipoprotein 21G1-like(LP21G1), microvitellogenin (MVG), and methionine aminopeptidase 2 (METAP2) before thermal induction; and histone 2A and nesprin-1 isoform X8 after thermal induction) for PRM validation (Supplementary Table S20). Although histone 2A and nesprin-1 were not successfully detected using PRM method, the PRM results of 13 other proteins showed a similar change in expression level with iTRAQ, indicating that the iTRAQ datasets are reproducible and reliable. The peptide quantification information of PRM analysis is provided in Supplementary Tables S21 and S22.

## 4. Discussion

Insects provide attractive models for the study of asexuality [38,39]. Comparative analysis between sexual and asexual reproduction has been conducted on different insect orders to uncover this transition [26,40,41]. The silkworm PL wu14 was originated from its parent AL 54A and reproduced parthenogenetically with genetic selection on the trait of high parthenogenetic incidence through thermal treatment. We speculated that the long time selection and the different reproductive mode would largely affect the expression of genes. Previous studies have revealed that parthenogenesis reproduction will affect the expression of sexually reproduced genes [38]. The difference of proteome profile between PL and AL before thermal induction will uncover the unique features of parthenogenetic reproduction. Although the transition of sexuality and asexuality remains uncovered, the common features between different insect orders may provide important information to better understand the molecular mechanism underlying parthenogenesis.

The comparative proteome before thermal induction showed that a total of 37 ribosomal proteins (RPs), such as RPL8/17/22/26, RPS5/8/9/11/25, etc., all showed increased abundance in PL, implying that protein synthesis may be enhanced in PL. Comparative transcriptomic analysis between obligately asexual and cyclically asexual rotifers also revealed that the expression of genes encoding ribosomal proteins is higher in OP lines than in CP lines [26]. Furthermore, five gene expression-related proteins, including small nuclear ribonucleoprotein G [42], U6 snRNA-associated Sm-like protein isoform X1 [43], small nuclear ribonucleoprotein Sm S2 [44], eukaryotic translation initiation factor 3 subunit G [45], and deoxyhypusine hydroxylase/monooxygenase [46], were increased in abundance in PL. We suggested that the increase in translation process might facilitate parthenogenetic development when subjected to thermal stimuli.

Many metabolism pathways including drug metabolism, ascorbate and aldarate metabolism, phenylalanine metabolism, tryptophan metabolism, and beta-alanine metabolism were enriched with increased abundance proteins before thermal induction, suggesting that many key metabolic proteins increased in PL. The same phenomenon is also found in *Daphnia pulex*. The genome-wide expression in OP females in comparison to CP females reveals that metabolic pathways are enriched with over-dominant genes [40]. Longevity is regulated by a network of closely linked metabolic systems [47]. The parthenogenetic reproduction mode seems to be favorable to the longevity of silkworm, for the abundance of related proteins including insulin-like peptide precursor, protein lethal (2), essential for life, superoxide dismutase [Cu-Zn], time interval-measuring enzyme-esterase A4, and heat shock proteins were increased in PL. However, our proteome data also revealed that the parthenogenesis reproduction mode may have negative effects on the immune response, since two immune-related pathways showed enrichment with decreased abundance proteins: NOD-like receptor signaling pathway and the C-type lectin receptor signaling pathway. This phenomenon has also been revealed in phasmatid (*Extatosoma tiaratum*). When parthenogenesis persists beyond one generation, it can have deleterious effects on the immune response in phasmatid [48]. The abundance of some development-related proteins were decreased in PL. Those proteins include chorions, calmodulin, histone H2A, and multiprotein bridging factor 1. The difference in developmental processes was also observed between OP and CP rotifer populations during asexual egg production [26]. So, there should be some common molecular mechanism of parthenogenetic development shared among different species.

The comparison of protein profiling patterns between PL and AL after thermal induction will illuminate the transition of meiosis to a parthenogenetic developmental process. In response to heat stress, cells will induce the transient expression of heat shock proteins (HSPs) [49,50]. Hsp70 and Hsc were increased in abundance after thermal induction in PL. The PPI analysis further revealed that Hsc may play an important role in response to the thermal stress in PL. Thermal treatment will result in protein unfolding in the silkworm oocyte; then, the endoplasmic reticulum (ER) homeostasis is disrupted for the accumulation of unfolded proteins in the ER [51]. To restore ER homeostasis, cells initiate the unfolded protein response (UPR) by decreasing protein synthesis, increasing degradation of unfolded proteins, and upregulating chaperone expression to enhance protein folding [52]. The PPI network based on the DAPs after thermal stimuli suggested an active UPR in PL. Furthermore, the increase in ascorbate and aldarate metabolism in the PL might promote the maintenance of redox homeostasis under high-temperature stress [53,54]. The increase of pyrimidine metabolism and increasing abundance of RAD50 may be a response to DNA damage [55,56]. The decrease in the thermogenesis pathway and oxidative phosphorylation mediates the regulation of energy metabolism, as well as the balance between heat production and dissipation during thermal treatment, which promotes adaptation to high-temperature environments [57]. It seems that PL has a more effective response to buffer thermal stress than AL.

Subsequent to thermal induction, most of the unfertilized eggs of the PL began to develop by parthenogenetic reproduction mode. For successful impaternate development, the thermal stimuli should trigger a procedure of oocyte at meiosis Ⅱ stage into mitosis and activate an early postmeiotic development [58]. Cytological investigation of the parthenogenetically activated eggs revealed complex rearrangements in the nuclei after thermal treatment, including the dissociation of bivalent chromosomes, the destruction of the spindle, and the formation of a new spindle and metaphase plate [58]. The spindle is a micron-sized chromosome segregation machine composed of microtubules and other proteins. Its assembly and functions are closely tied to the dynamics of microtubules [59]. In the present study, no significant differences in the abundance of α-, β-, and γ- tubulin were observed between the PL and AL. However, the microtubule-associated protein futsch, which controls the stability of microtubules, was decreased [60]. Three actin proteins were also decreased in abundance in the PL Wu 14. Actin cortex functions in the processes of spindle assembly and guiding spindle orientation with respect to extracellular chemical and mechanical cues [61]. Further, the largest fold changes occurred in a set of other cytoskeleton proteins, including nesprin, tropomyosin, troponin I, and myosin in the PL. Nesprin mediates the establishment of centrosome localization [62]. Tropomyosin mediates the regulation of cytoplasmic myosins [63], and myosin is involved in meiotic spindle disassembly [64]. Troponin I takes a role in nuclear division [65]. The decreased abundance of those cytoskeleton-related proteins might be responsible for the rearrangement of spindle assembly during the ameiotic process of parthenogenetic activation.

The investigation in *Daphnia pulex* indicates that the transition from meiosis to parthenogenesis requires at least three modifications: changes in the spindle attachment of the kinetochore, modification of sister chromatid cohesion, and abrogation of homologous recombination [41,66]. In the present study, an uncharacterized protein which is homologous to the kinetochore protein Ndc80 was decreased in abundance in PL. Kinetochores control chromosome segregation by mediating their interaction with spindle microtubules during mitosis and meiosis [67]. The Ndc80 complex contains a contact point for microtubules [68,69]. We then speculate that the decrease in Ndc80 might contribute to the changes in the spindle attachment of the kinetochore during the parthenogenesis activation in the PL. Chromatin condensation and compaction are essential for accurate chromosome segregation during cell division [70]. Histones (histone H2A, H2B-like, histone H3-like, histone H4, and histone H1-like) all showed decreased abundance in PL; this decrease may be involved in chromatin dynamics in the ameiotic processes.

When undergoing the sexual reproduction mode, a mature oocyte is activated by sperm [71]. For parthenogenetic reproduction, it must be able to trigger egg activation without sperm [72]. Proteomic analyses have been used to characterize changes in the abundance of proteins in *Drosophila* embryos during the oocyte-to-embryo transition [29,73,74]. In fertilized eggs, nearly all animals studied to date shared the fact of calcium rise, and thus, egg activation [75]. However, insects have a unique mode where fertilization is not necessary for egg activation [76]. Recent studies in *Drosophila* revealed that egg activation requires calcium and that the downstream events and molecules of egg activation are also conserved, despite the difference in initial trigger [75]. Phospholipase C (PLC) has the activity of generating IP3 to release Ca^2+^ [77], playing a role in egg activation [78]. In the present study, the abundance of phosphatidylinositol phospholipase C, gamma-1, was increased in PL after parthenogenetic activation, indicating a possible increase in Ca^2+^ in PL after thermal induction, which may be also a necessary event in triggering parthenogenesis development. 

In the sexual reproduction mode, the oocyte-to-embryo transition is marked by a change in redox state [79]. The dynamic redox balance directs the oocyte-to-embryo transition via developmentally controlled reactive cysteine changes [79]. ROS are mainly derived from oxidative phosphorylation in the mitochondria [80]. In the current study, the GO enrichment analysis showed that 12 DAPs involved in the oxidation-reduction process were decreased after thermal induction. Thioredoxin-related transmembrane protein 1 (TMX1) and thioredoxin domain-containing protein 2 isoform X2, were also decreased in the PL following parthenogenetic activation. TMX1 regulates the homeostasis of the endoplasmic reticulum during post-stress recovery [81]. The decreased abundance of DAPs related to oxidation and reduction processes, and thioredoxin-related proteins suggest a redox-modulated control in the PL, which might play a key role in the oocyte-to-embryo transition after thermal induction. ROS level was also found to be related to permitting the progression of apomeiosis [82].

The oocyte’s outer coverings are specialized to allow sperm binding and entry in sexual reproduction. Changes and modifications in egg coverings play key roles in egg activation [83]. Following thermal induction, 14 chorion proteins were differentially expressed, and 13 were increased in abundance in PL, such as chorion class B protein L12-like, chorion class B protein M2410-like, and chorion class A protein M2774-like, suggesting their role in egg activation. Tyrosine and dopamine are major precursors for synthesis of black melanin and N-acylquinoid-derived pigments [84]. Dopachrome tautomerase is an enzyme responsible for melanisation in egg chorion tanning [85]. We found that dopachrome tautomerase was increased in abundance in PL. The increased abundance of chorion proteins and dopachrome tautomerase seems to be an indicator for successful parthenogenesis activation since, when the egg is activated, it will get to tanning during embryo development.

## 5. Conclusions

In the present study, we identified a set of proteins with significantly higher expression in unfertilized eggs of either the parthenogenetic line (PL) or its parent amphigenetic line (AL) of silkworm, *Bombyx mori*. Included in this set of proteins are those with potential roles in processes specific to thermal-induced parthenogenesis, such as thermal stress response, cell cycle, and spindle assembly. The functional annotation of those DAPs before thermal induction revealed differences in translation, metabolism, and development processes, which seems to have a molecular basis associated with the consequences of different reproductive modes and parthenogenesis capacity between PL and AL. Furthermore, although some key proteins and pathways have been uncovered, many mechanisms need to be investigated further, such as how the thermal stimuli regulate the ameiosis process. To illuminate this question, not enough hints were provided by the present study. The identification of relevant receptors and analysis of signal transduction pathways will be carried out in our further investigation.

## Figures and Tables

**Table 1:** The cell cycle-related differential abundance proteins (DAPs) after thermal induction.

Accession Number	Protein Description	Fold Change	*p*-Value	Function
Bmb007646	serine/threonine-protein kinase Warts	1.35	9.45 × 10^−3^	Cell cycle control, cell division, chromosome partitioning
ref|XP_021203402.1|	uncharacterized protein LOC101735506	0.76	6.41 × 10^−3^	Cell cycle control, cell division, chromosome partitioning
ref|XP_004930078.1|	lamin-C	0.78	2.76 × 10^−2^	Cell cycle control, cell division, chromosome partitioning; nuclear structure
ref|XP_004934091.1|	histone H3-like, partial	0.53	1.28 × 10^−3^	Chromatin structure and dynamics
ref|NP_001153666.1|	histone H2A	0.53	2.30 × 10^−3^	Chromatin structure and dynamics
ref|XP_004934126.1|	histone H1-like, partial	0.64	3.23 × 10^−3^	Chromatin structure and dynamics
ref|NP_001153669.1|	histone H4	0.6	7.54 × 10^−3^	Chromatin structure and dynamics
ref|XP_004934078.2|	histone H2B-like, partial	0.58	1.22 × 10^−2^	Chromatin structure and dynamics
ref|XP_021208330.1|	bromodomain adjacent to zinc finger domain protein 2B isoform X8	3.63	2.37 × 10^−2^	Replication, recombination, and repair
ref|XP_021207651.1|	DNA repair protein RAD50	4.11	2.37 × 10^−2^	Replication, recombination, and repair
Bmb004885	oxygen resistance gene 1 isoform X9	0.71	2.67 × 10^−2^	Replication, recombination, and repair
ref|XP_004922235.1|	protein artemis-like isoform X1	0.65	3.48 × 10^−2^	Replication, recombination, and repair

**Table 2:** The cytoskeletal protein expression pattern in PL after thermal induction.

Accession Number	Protein Description	Fold Change	*p*-Value
ref|XP_004928315.1|	alpha-actinin, sarcomeric isoform X1	0.44	1.15 × 10^−5^
ref|NP_001124374.1|	paramyosin	0.42	4.57 × 10^−5^
ref|XP_021202926.1|	microtubule-associated protein futsch	0.35	5.14 × 10^−3^
ref|NP_001091813.1|	myosin regulatory light chain 2	0.31	1.12 × 10^−4^
ref|NP_001103782.1|	tropomyosin-2 isoform 1	0.31	1.15 × 10^−4^
Bmb003576	nesprin-1 isoform X8	0.31	1.21 × 10^−4^
ref|XP_021203153.1|	myosin heavy chain, muscle-like	0.31	1.39 × 10^−4^
ref|XP_012545977.1|	troponin T isoform X1	0.32	3.76 × 10^−4^
ref|NP_001037295.1|	troponin I	0.33	4.69 × 10^−4^
ref|NP_001040445.1|	tropomyosin-1	0.48	1.20 × 10^−3^
ref|NP_001119724.1|	actin, muscle-type A1	0.48	1.61 × 10^−3^
ref|XP_012549671.1|	myosin light chain alkali	0.39	2.02 × 10^−3^
ref|XP_012548582.1|	nesprin-1 isoform X3	0.66	3.20 × 10^−3^
ref|NP_001119725.1|	actin, muscle-type A2	0.49	5.96 × 10^−3^
ref|XP_012550186.1|	tropomodulin-1 isoform X7	0.68	1.56 × 10^−2^
ref|NP_001040476.1|	muscular protein 20	0.63	1.79 × 10^−2^
ref|XP_021209322.1|	twitchin	0.82	2.26 × 10^−2^
ref|XP_004929461.1|	myophilin	0.69	2.88 × 10^−2^

**Table 3:** Gene ontology (GO) enrichment analysis for increased abundance proteins in PL before thermal induction.

GO ID	GO Terms	Category	*p* Value
GO:0005840	ribosome	C	4.31 × 10^−12^
GO:0030529	intracellular ribonucleoprotein complex	C	2.13 × 10^−11^
GO:1990904	ribonucleoprotein complex	C	2.13 × 10^−11^
GO:0043228	non-membrane-bounded organelle	C	7.64 × 10^−7^
GO:0043232	intracellular non-membrane-bounded organelle	C	7.64 × 10^−7^
GO:0044444	cytoplasmic part	C	3.73 × 10^−5^
GO:0032991	macromolecular complex	C	1.27 × 10^−3^
GO:0044391	ribosomal subunit	C	1.72 × 10^−3^
GO:0043226	organelle	C	3.16 × 10^−3^
GO:0043229	intracellular organelle	C	3.16 × 10^−3^
GO:0003735	structural constituent of ribosome	F	2.35 × 10^−11^
GO:0005198	structural molecule activity	F	7.31 × 10^−5^
GO:0019843	rRNA binding	F	1.36 × 10^−3^
GO:0016491	oxidoreductase activity	F	2.75 × 10^−2^
GO:0003857	3-hydroxyacyl-CoA dehydrogenase activity	F	3.73 × 10^−2^
GO:0070006	metalloaminopeptidase activity	F	3.73 × 10^−2^
GO:0004497	monooxygenase activity	F	4.97 × 10^−2^
GO:0006412	translation	P	8.41 × 10^−7^
GO:0043043	peptide biosynthetic process	P	8.41 × 10^−7^
GO:0043604	amide biosynthetic process	P	8.41 × 10^−7^
GO:0010467	gene expression	P	8.47 × 10^−7^
GO:0006518	peptide metabolic process	P	1.25 × 10^−6^
GO:0043603	cellular amide metabolic process	P	1.83 × 10^−6^
GO:1901566	organonitrogen compound biosynthetic process	P	1.82 × 10^−5^
GO:0044267	cellular protein metabolic process	P	2.57 × 10^−5^
GO:0044271	cellular nitrogen compound biosynthetic process	P	5.03 × 10^−5^
GO:0009059	macromolecule biosynthetic process	P	1.33 × 10^−4^

**Table 4:** GO enrichment analysis for increased abundance proteins in PL after thermal induction.

GO ID	GO Terms	Category	*p* Value
GO:0030312	external encapsulating structure	C	3.59 × 10^−15^
GO:0042600	chorion	C	3.59 × 10^−15^
GO:0071944	cell periphery	C	3.32 × 10^−12^
GO:0005213	structural constituent of chorion	F	1.25 × 10^−15^
GO:0005198	structural molecule activity	F	1.85 × 10^−6^
GO:0016757	transferase activity, transferring glycosyl groups	F	2.10 × 10^−2^
GO:0002064	epithelial cell development	P	4.28 × 10^−14^
GO:0002065	columnar/cuboidal epithelial cell differentiation	P	4.28 × 10^−14^
GO:0002066	columnar/cuboidal epithelial cell development	P	4.28 × 10^−14^
GO:0007304	chorion-containing eggshell formation	P	4.28 × 10^−14^
GO:0030703	eggshell formation	P	4.28 × 10^−14^
GO:0030707	ovarian follicle cell development	P	4.28 × 10^−14^
GO:0030855	epithelial cell differentiation	P	4.28 × 10^−14^
GO:0009888	tissue development	P	1.37 × 10^−13^
GO:0048477	oogenesis	P	1.37 × 10^−13^
GO:0060429	epithelium development	P	1.37 × 10^−13^

**Table 5:** Validation of differential abundance proteins (DAPs) with parallel reaction monitoring (PRM analysis).

Pathway	Protein ID	Protein Description	Comparison	iTRAQ Ratio	iTRAQ *p*-Value	PRM Ratio	PRM *p*-Value
Ribosome	ref|NP_001037275.1|	ribosomal protein S25	PL-uninduced/AL-uninduced	1.27	0.000146	1.61	1.48 × 10^−3^
	ref|NP_001037225.1|	ribosomal protein L22	PL-uninduced/AL-uninduced	1.25	1.21 × 10^−6^	1.33	5.39 × 10^−2^
	ref|NP_001037233.1|	ribosomal protein L26	PL-uninduced/AL-uninduced	1.72	5.22 × 10^−7^	1.32	6.24 × 10^−2^
	ref|NP_001037141.1|	ribosomal protein L8	PL-uninduced/AL-uninduced	2.05	1.11 × 10^−5^	1.36	7.63 × 10^−2^
	ref|NP_001037165.1|	60S ribosomal protein L17	PL-uninduced/AL-uninduced	2.48	0.000023	1.34	8.01 × 10^−2^
Thermogenesis	ref|NP_001040428.1|	H+ transporting ATP synthase gamma subunit	PL-induced/AL-induced	0.71	7.89 × 10^−5^	0.70	1.05 × 10^−2^
	ref|XP_004933701.1|	NADH dehydrogenase [ubiquinone] iron-sulfur protein 3, mitochondrial	PL-induced/AL-induced	0.64	0.0013	0.62	3.26 × 10^−2^
	ref|XP_004933852.2|	electron transfer flavoprotein subunit alpha, mitochondrial	PL-induced/AL-induced	0.67	0.000989	0.83	8.70 × 10^−2^
	ref|XP_004932565.1|	Probable aconitate hydratase, mitochondrial	PL-induced/AL-induced	0.67	0.003989	0.71	1.39 × 10^−1^
	ref|NP_001296504.1|	cytochrome c	PL-induced/AL-induced	0.33	0.000272	0.46	1.63 × 10^−1^

## Data Availability

Data are contained within the article or supplementary material.

## Supplementary Materials

The following supporting information can be downloaded at: https://www.mdpi.com/article/10.3390/insects14020134/s1, Supplementary Table S1. Statistics of parthenogenesis incidence of PL Wu14 and AL 54A after thermal induction; Supplementary Table S2. All identified proteins in PL/AL before thermal induction; Supplementary Table S3. All identified proteins in PL/AL after thermal induction; Supplementary Table S4. The significant DAPs in PL/AL before thermal induction; Supplementary Table S5. The significant DAPs in PL/AL after thermal induction; Supplementary Table S6. The overlapping DAPs before and after thermal induction; Supplementary Table S7. Subcellular location of the increased abundance proteins in PL/AL before thermal induction; Supplementary Table S8. Subcellular location of the decreased abundance proteins in PL/AL before thermal induction; Supplementary Table S9. Subcellular location of the increased abundance proteins in PL/AL after thermal induction; Supplementary Table S10. Subcellular location of the decreased abundance proteins in PL/AL after thermal induction; Supplementary Table S11. GO enrichment for the increased abundance proteins in PL/AL before thermal induction; Supplementary Table S12. GO enrichment for the decreased abundance proteins in PL/AL before thermal induction; Supplementary Table S13. GO enrichment for the increased abundance proteins in PL/AL after thermal induction; Supplementary Table S14. GO enrichment for the decreased abundance proteins in PL/AL after thermal induction; Supplementary Table S15. KEGG pathway enrichment analysis for the increased abundance proteins in PL/AL before thermal induction; Supplementary Table S16. KEGG pathway enrichment analysis for the decreased abundance proteins in PL/AL before thermal induction; Supplementary Table S17. KEGG pathway enrichment analysis for the increased abundance proteins in PL/AL after thermal induction; Supplementary Table S18. KEGG pathway enrichment analysis for the decreased abundance proteins in PL/AL after thermal induction; Supplementary Table S19. The DAPs mapped to the protein–protein interactions; Supplementary Table S20. PRM validation for random selected DAPs; Supplementary Table S21. The peptide intensity of PRM validation for DAPs of ribosome pathway and random selected before thermal induction; Supplementary Table S22. The peptide intensity of PRM validation for DAPs of thermogenesis pathway after thermal induction. Supplementary Figure S1. The response of oxidative phosphorylation pathway after thermal induction in parthenogenetic line. The red rectangle indicates upregulated proteins and the green one indicates downregulated proteins; Supplementary Figure S2. The response of citrate cycle pathway after thermal induction in parthenogenetic line; the red rectangle indicates upregulated proteins and the green one indicates downregulated proteins. Supplementary Figure S3. The response of Hippo signaling pathway after thermal induction in parthenogenetic line. The red rectangle indicates upregulated proteins and the green one indicates downregulated proteins; Supplementary Figure S4. The response of ECM–receptor interaction pathway after thermal induction in parthenogenetic line. The red rectangle indicates upregulated proteins and the green one indicates downregulated proteins.
