# Supplementary material for: Comparative Proteomic Analysis Provides New Insights into the Molecular Basis of Thermal-Induced Parthenogenesis in Silkworm (Bombyx mori)
Source: Insects. 2023 Jan 28;14(2):134. doi: 10.3390/insects14020134 (PMC9962255; doi:10.3390/insects14020134)
Supplement: Supplementary file 1 [file insects-14-00134-s001.zip › Supplementary Figures.pdf]

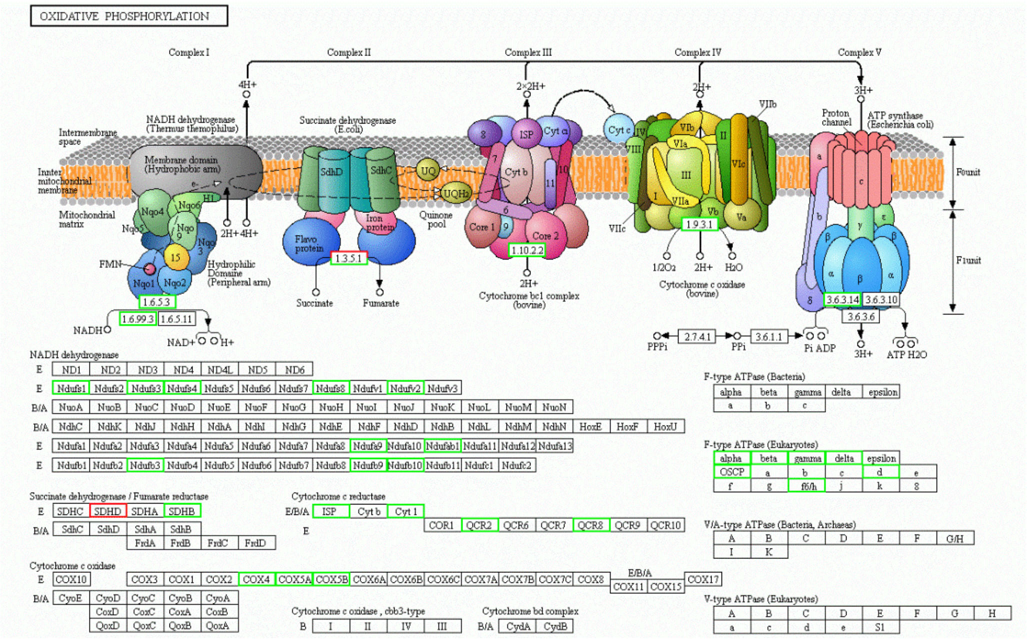

Supplementary Figure S1. The response of oxidative phosphorylation pathway after thermal induction in parthenogenetic line. The red rectangle indicates up-regulated proteins and the green one indicates down-regulated proteins.

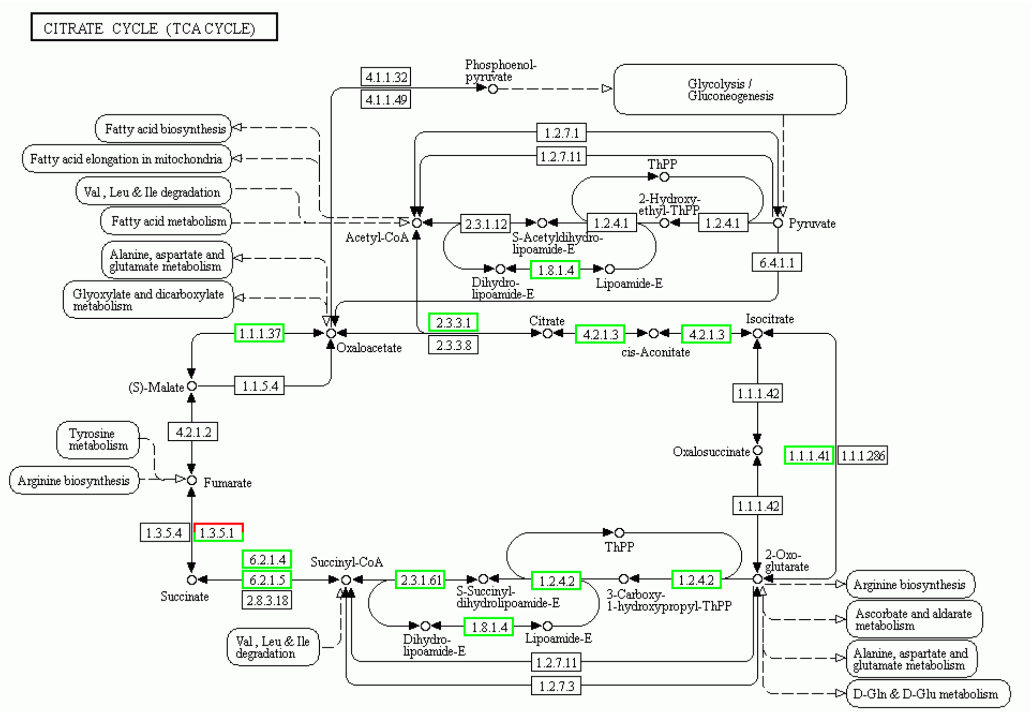

Supplementary Figure S2. The response of citrate cycle pathway after thermal induction in parthenogenetic line. The red rectangle indicates up-regulated proteins and the green one indicates down-regulated proteins.

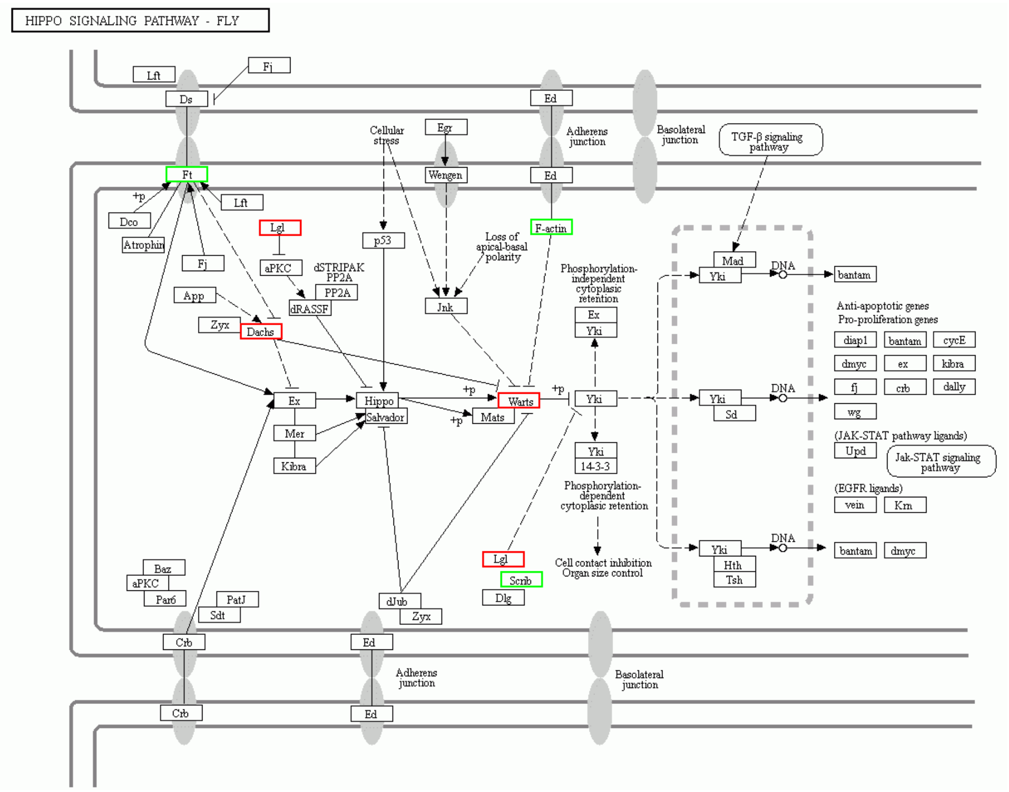

Supplementary Figure S3. The response of Hippo signaling pathway after thermal induction in parthenogenetic line. The red rectangle indicates up-regulated proteins and the green one indicates down-regulated proteins.

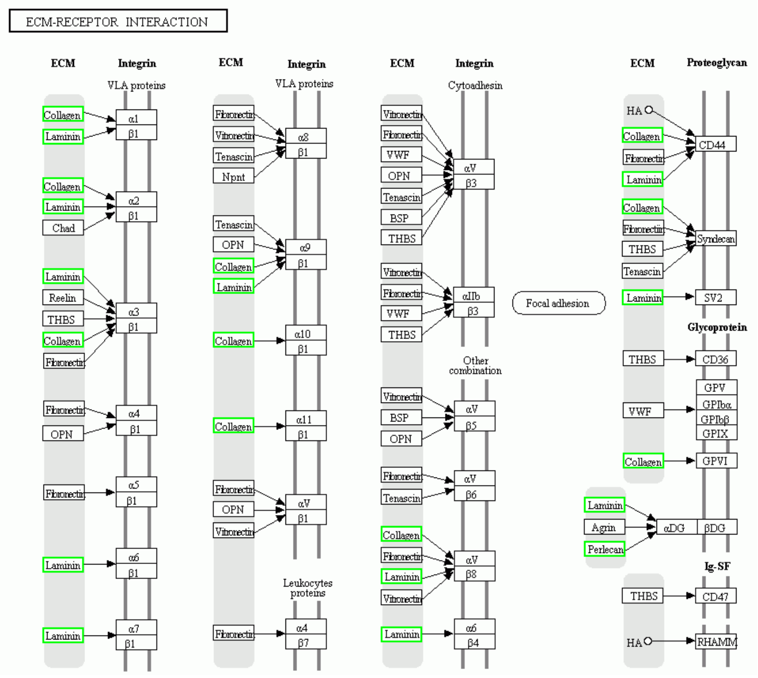

Supplementary Figure S4. The response of ECM-receptor interaction pathway after thermal induction in parthenogenetic line. The red rectangle indicates up-regulated proteins and the green one indicates down-regulated proteins.
